# Visual awareness negativity is an early neural correlate of awareness: A preregistered study with two Gabor sizes

**DOI:** 10.3758/s13415-018-0562-z

**Published:** 2018-01-18

**Authors:** Rasmus Eklund, Stefan Wiens

**Affiliations:** Psykologiska Institutionen, Stockholm University, Stockholm, Sweden

**Keywords:** Visual awareness, Event-related potentials, Visual awareness negativity, Late positivity, Gabor size

## Abstract

Electrophysiological recordings are commonly used to study the neural correlates of consciousness in humans. Previous research is inconsistent as to whether awareness can be indexed with visual awareness negativity (VAN) at about 200 ms or if it occurs later. The present study was preregistered with two main aims: First, to provide independent evidence for or against the presence of VAN, and second, to study whether stimulus size may account for the inconsistent findings. Subjects were shown low-contrast Gaussian filtered gratings (Gabor patches) in the four visual quadrants. Gabor size (large and small) was varied in different sessions and calibrated to each subject’s threshold of visual awareness. Event-related potentials were derived from trials in which subjects localized the Gabors correctly to capture the difference between trials in which they reported awareness versus no awareness. Bayesian analyses revealed very strong evidence for the presence of VAN for both Gabor sizes. However, there was no evidence for or against an effect of stimulus size. The present findings provide evidence for VAN as an early neural correlate of awareness.

The phenomenon and mechanism of aware visual experiences are still debated and remain largely unclear (Block, 2007; Dehaene & Changeux, 2011; Lamme, 2010; Tononi, Boly, Massimini, & Koch, 2016). As a first step towards establishing the neural mechanism of awareness in humans, research has focused primarily on the neural correlates of visual awareness. Crick and Koch (1998) advised to start with visual awareness because visual input is highly structured and easy to manipulate, and many aspects of the neural mechanisms of vision are well understood from animal research. In this study, we use visual awareness to refer to phenomenal consciousness: “what it is like” to have an experience (Block, 1995) comparable to the quality of the experience (Revonsuo, 2006) or qualia (Dennett, 1988). We investigate the content of visual awareness, the experience of seeing a transient stimulus, which is notably different from sustained visual awareness or states of consciousness (Andersen, Pedersen, Sandberg, & Overgaard, 2016).

Visual awareness is manipulated by changing the visibility of a stimulus and is assessed using subjective and objective measures. A commonly used objective measure is a forced-choice response, which results in either the correct or incorrect detection of the stimulus. A commonly used subjective measure is a self-reported rating of the subject’s awareness of the stimulus. Based on one or both of these measures, trials are classified as *aware* and *unaware*. When brain activity is compared between aware trials and unaware trials, differences in neural activity between these trials are considered the neural correlates of visual awareness (Crick & Koch, 1998). To avoid confounds between visual perception and stimulus properties, it is essential that the physical properties of visual stimuli (e.g., contrast, luminance) be the same on aware and unaware trials (Frith, Perry, & Lumer, 1999).

Research on visual evoked potentials derived from electroencephalography (EEG) has suggested two potential neural correlates of visual awareness: visual awareness negativity (VAN) and late positivity (LP; for a review, see Koivisto & Revonsuo, 2010). With respect to VAN, research has shown that the N200, a negative-going peak at posterior, occipital electrodes present about 200 ms after visual stimulus onset (thus, in the range of N1 and N2) differs as a function of visual awareness. Specifically, the N200 is larger for stimuli reported as aware than for stimuli reported as unaware, and therefore this relative negativity is referred to as VAN (Koivisto & Revonsuo, 2010; Ojanen, Revonsuo, & Sams, 2003). Research on visual awareness has also implicated the P3, which is the third major positivity at parietal electrodes present after about 300 ms. This work suggests that the P3 is more positive for stimuli reported as aware versus unaware, and this relative positivity is referred to as LP (Wilenius & Revonsuo, 2007). Evidence for one or both of these components has been found across a variety of methods that manipulate visual awareness, including masking, attentional blink, change blindness, manipulation of contrast, and bistable perception (for a review, see Koivisto & Revonsuo, 2010).

Current theories disagree on the meaning of VAN and LP in the context of visual awareness. According to the recurrent processing theory (Lamme, 2010; Lamme & Roelfsema, 2000), feedback processing in sensory cortex (local recurrent processing) enables phenomenal consciousness, whereas feedback processing between sensory and frontal-parietal association cortex (global recurrent processing) enables access consciousness. In contrast to phenomenal consciousness, which involves only the quality of the experience, access consciousness is the state of introspecting or reporting about an experience (Block, 1995). In line with recurrent processing theory (Andersen et al., 2016; Koch, Massimini, Boly, & Tononi, 2016; Koivisto, Salminen-Vaparanta, Grassini, & Revonsuo, 2016), VAN is thought to reflect local recurrent processing; thus, it should be an index of phenomenal consciousness. On the other hand, LP is thought to reflect global recurrent processing; thus, it should be an index of access consciousness.

In contrast, the global neuronal workspace theory suggests that VAN is not a neural correlate of awareness but rather reflects unconscious processing (Dehaene, Changeux, Naccache, Sackur, & Sergent, 2006). Further, in this view, the widespread global activation or ignition of the prefrontoparietal network that gives rise to visual awareness is reflected by LP (Dehaene & Changeux, 2011). Thus, proponents of the recurrent processing theory (*early* theory) argue that VAN ought to be a robust correlate of visual awareness. In contrast, proponents of the global neuronal workspace theory (*late* theory) argue that VAN is merely a prerequisite for global activation but does not necessarily correlate with visual awareness (Lamy, Salti, & Bar-Haim, 2009).

In support of the early theory, previous research found that VAN correlated with visual awareness (for a review, see Koivisto & Revonsuo, 2010). However, because many of these studies only used subjective measures to index awareness, they are susceptible to potential confounds because of individual differences in performance (for a review, see Lamy et al., 2009). Specifically, if only a subjective measure is used to define awareness, the aware trials may be mostly correct trials, whereas unaware trials may be mostly incorrect trials, thus confounding the effect of awareness with differences in performance.

Two recent studies that controlled for performance challenged the idea that VAN is the earliest correlate of visual awareness (Lamy et al., 2009; Salti, Bar-Haim, & Lamy, 2012). In these studies, only trials that were correctly localized were considered, and these correct trials were categorized into aware and unaware, according to subjective reports. In the Lamy et al. (2009) study, subjects were shown backward-masked line segments in the four quadrants of the visual field. After the stimulus, subjects responded to the location of the target and rated their subjective experience with two responses: they saw the target or they guessed its location. When subjects rated that they were guessing, performance was well above chance (between 50% and 60% correct, even though 25% was chance level). According to the authors, unaware correct trials were above chance because they comprised two types of trials: those that were correct by chance and those that underwent enough unconscious processing to elicit a correct response. To adjust for the effect of chance, the ERP to unaware incorrect trials was used to obtain a “chance-free” estimate of the ERP to unaware correct trials. This adjusted ERP ought to represent correct responses from unconscious processing alone. Therefore, the aware-correct ERP minus the chance-free unaware-correct ERP should capture conscious visual processing alone (Lamy et al., 2009). The results showed a larger P3 for aware-correct than for unaware-correct trials, but there was no difference for N1, N2, P1, or P2. These findings provide evidence that LP, but not VAN, is an index of visual awareness.

Salti et al. (2012) used the same method as Lamy et al. (2009) but controlled for confidence as well as performance. When rating awareness, subjects used three levels: certain they had seen the target, not sure whether they had seen the target, or certain they had not seen the target. Trials in the middle rating category (i.e., not sure) were excluded. Results showed that aware-correct trials minus unaware-correct trials showed a larger positivity over parietal electrodes for P3 (i.e., LP). No difference was found for N1 or N2 at any electrodes. Because these two studies found evidence for LP but not VAN, the authors concluded that LP was the earliest correlate of visual awareness (Lamy et al., 2009; Salti et al., 2012).

In contrast, Koivisto and Grassini (2016) argued that VAN may not have been found in the previous studies (Lamy et al., 2009; Salti et al., 2012) for several reasons: (1) Small stimulus size (1.5°) may have activated only a small neural population; (2) backward masking may have resulted in a summed response to target and mask; and (3) pooling across contralateral and ipsilateral stimulation may have decreased sensitivity, as VAN should occur in a retinotopic manner contralateral rather than ipsilateral to the stimulated visual hemifield (Koivisto & Revonsuo, 2010). For these reasons, the design in previous studies may have been insensitive to detect VAN.

To address these issues, Koivisto and Grassini used large (3.8°) Gaussian-filtered gratings (Gabors) that were presented at low contrast rather than backward masked, and contralateral and ipsilateral electrodes were analyzed separately. Critically, Koivisto and Grassini found evidence for VAN as well as LP. As such, these findings supported their claim that VAN, rather than LP, is the earliest ERP correlate of visual awareness. In an additional analysis, the mean N200 was computed for each subject across unaware correct trials and across unaware incorrect trials. Across subjects, a larger N200 difference of unaware correct trials minus unaware incorrect trials correlated with a more conservative response criterion (i.e., signal-detection index *C*). This suggests that the N200 may have been enhanced for subjects who may have been weakly aware of the stimuli but were unwilling to report awareness, that is, they rated trials as unaware rather than as aware (Koivisto & Grassini, 2016). If so, comparing aware and unaware correct trials may underestimate the neural effect of visual awareness, and therefore VAN would be a conservative measure of visual awareness.

Because previous studies that controlled for subjects’ objective performance reported inconsistent results (Koivisto & Grassini, 2016; Lamy et al., 2009; Salti et al., 2012), the present study aims to replicate and extend this work. We chose to use the design employed by Koivisto and Grassini (2016) because it appeared to be a promising task to observe VAN. However, because none of the previous studies controlled for physical differences between the quadrants, results may be confounded by differences in the physical stimuli. For example, if aware correct trials were mainly trials in the upper right quadrant and unaware correct trials were mainly trials in the lower left quadrant, any differences in the ERPs may be caused by the different quadrants rather than by the subjects’ responses. Such confounds may be anticipated. For example, upper visual field stimulation results in smaller N1 (between about 120 and 220 ms) than does lower visual field stimulation (Capilla et al., 2016). Therefore, we controlled for this potential confound by computing ERPs separately for each quadrant before averaging across the quadrants. Furthermore, to increase the evidential strength of our results and reduce “researcher degrees of freedom,” we preregistered our methods and analyses in as much detail as possible (Eklund & Wiens, 2017, osf.io/4a4ur). This is particularly relevant for ERP studies because the large number of electrodes that are recorded for many time samples result in a multiple comparison problem that is prone to yield false positives, particularly in factorial designs (Luck & Gaspelin, 2017). Last, because most labs use computers with Windows operating systems that do not allow for precise timing, photodiode recordings of the Gabor onsets and offsets were used to define the exact onset of the Gabors and to validate their duration. Because ERPs were time locked to the photodiode recordings, this procedure eliminated any latency jitters and thus maximized sensitivity in detecting VAN and LP.

Another main purpose of the study was to address the suggestion by Koivisto and Grassini (2016) that large stimuli result in a large VAN. In line with Bullier (2001), processing in V1 and V2 is modulated by recurrent feedback from higher visual areas. Thus, V1 and V2 act as “active blackboards” enabling visual awareness by integrating information from higher visual areas. Because a large stimulus activates larger areas of V1 and V2, recurrent processing ought to be increased. Accordingly, the larger the stimulus and its corresponding activation in V1 and V2, the larger the recurrent feedback and the larger the VAN. In the present study, subjects completed two experimental sessions, and we varied Gabor size between sessions (large at 3.8° and small at 1.5°). The size of the large Gabor was identical to that in Koivisto and Grassini (2016). The size of the small Gabor approximated the size of the visual stimuli used by Lamy et al. (2009) and Salti et al. (2012).

## Method

### Participants

The initial sample consisted of 35 healthy subjects (*M* = 26.29 years, *SD* = 4.65), of which 12 were males and 29 right-handed. All subjects had normal or corrected-to-normal vision and were recruited from local universities and through online billboards. They were compensated with either movie vouchers or course credits after their participation in two sessions (large Gabor and small Gabor). All subjects participated in both sessions. Before each session, subjects provided written consent in accordance with the Declaration of Helsinki. The research was conducted in accordance with the principles of the regional ethics board.​

When the preregistered exclusion criteria were adopted, several subjects were excluded from one of the sessions: Subjects did not correctly localize 70% of the control trials (*n* = 1 for large Gabors and *n* = 7 for small Gabors). Subjects did not show any clear N200 in the control trials (*n* = 4 for large Gabors and *n* = 6 for small Gabors). Specifically, for each subject, we inspected the ERP across control trials that were correct and reported as seen. A subject was excluded if no negativity (i.e., N200) was apparent between 180 and 280 ms across O1 and O2. Notably, these intervals and electrodes showed a clear N200 in the grand mean ERP across subjects (as described below). Critically, this decision occurred before analyzing each subject’s ERPs to the critical trials. Further, subjects were excluded if they had fewer than five critical trials per quadrant (*n* = 7 for large Gabors and *n* = 13 for small Gabors), as explained below. The final samples comprised 24 subjects in the session with the large Gabor (nine males; *M*_age_ = 26.30, *SD* = 4.50), 15 subjects in the session with the small Gabor (five males; *M*_age_ = 26.20, *SD* = 3.59), and 13 subjects who completed both sessions.

### Apparatus and stimuli

Stimuli were low-contrast sinusoidal Gabor patches (10 cycles/degree, vertically oriented). Gabor size was large (at 3.8° in diameter) and small (at 1.5°). Gabors were shown in one of the four visual quadrants: Large Gabors had their border at 0.3° from the fixation cross, and small Gabors had their border at 1.45° from the fixation cross. The centers of both Gabors were located 2.2° from the fixation cross.

Gabors were shown on a BenQ XL2430T, 24-inch gaming monitor (at 144 Hz, 1920 × 1080 resolution). PsychoPy Version 1.84 (Peirce, 2007) was used to generate and present stimuli, and to collect behavioral data. Because computers with a Windows operating system were used to present the Gabors, we anticipated timing errors between the actual presentation of the Gabors and their event markers from the presentation computer. To compensate for these timing errors, Gabor onsets and offsets were detected with two photodiodes attached in the corners of the screen. Simultaneously with Gabor onset, one white square was presented under one photodiode, and simultaneously with Gabor offset, another white square was presented under the other photodiode. Together with the event markers from the presentation computer, these signals were recorded as TTL triggers with a Cedrus StimTracker (Cedrus Corporation, San Pedro, CA).

### Procedure

Subjects completed two separate sessions (scheduled for the same time on different days), one with the large Gabor and one with the small Gabor (mean number of days between sessions = 7, ranging from 2 to 30 days). Gabor size was pseudorandomized over consecutive subjects; that is, within a set of six subjects, Gabor size for the first session was randomly sampled from three large and three small Gabor sizes. Notably, the experimenter was blind to Gabor size. Other than Gabor size (large or small), the procedure was identical in both sessions. In each session, subjects performed a detection task that comprised 580 trials (400 critical, 100 catch, and 80 control) in random order for each subject. *Critical* trials showed a Gabor for a calibrated duration (see below), *catch* trials were blank for the calibrated duration, and *control* trials showed a Gabor for 119 ms. For both critical and control trials, an equal number of Gabors were randomly assigned to the four quadrants. Trials were divided into five blocks of 116 trials with short breaks between blocks.

**Fig. 1:**
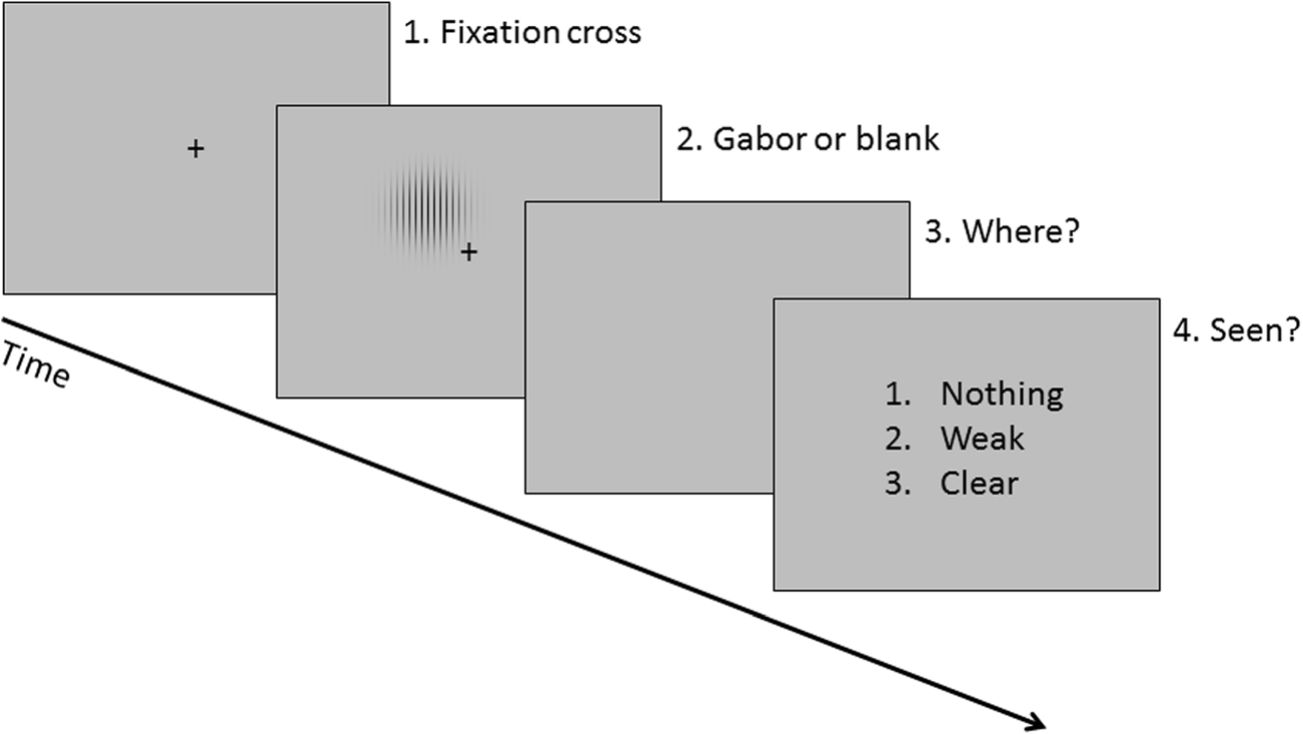
Time course of a trial

Figure 1 shows the time course of a trial. On each trial, a black fixation cross (0.1°) was displayed on a gray background for 1,200 ms followed by a Gabor or blank. The Gabor appeared in one of the four visual quadrants. The fixation cross was shown for the duration of the stimulus. Afterwards, subjects responded about stimulus location (four buttons corresponding to the quadrants; that is, insert, page up, delete, and page down on a computer keyboard). Last, they rated subjective visual awareness of the stimulus (“I did not see any stimulus,” “I saw the stimulus weakly,” or “I saw the stimulus clearly”) with three buttons (1, 2, and 3, respectively). Responses were nonspeeded.

Before the experiment, subjects started with a short practice task that was identical to the experimental task but with visible Gabors (119-ms duration and higher contrast). Then, an interleaved staircase (i.e., three separate staircases starting from a Gabor duration of 119, 63, or 14 ms) was used to calibrate the duration of the critical low-contrast Gabor to be reported as seen at approximately 50% of the trials. When the subject reported seeing the stimulus, the duration decreased. When the subject reported not seeing the stimulus, the duration increased. For each staircase, step sizes for the first five reversals were 28, 28, 14, 14, and 7 ms, and step size was 7 ms for the remaining reversals. In total, the staircase comprised 84 trials (28 trials per staircase). The calibrated duration was the mean of the last six reversals. After the calibration, a validation block was run (same procedure as in the experiment, but with 52 trials). If between 45% and 55% of the trials were reported as seen, the experiment began. If not, the Gabor duration was adjusted for another validation block. Validation was repeated for no more than five blocks until the 50% criterion was met. Even if the validation criterion was not reached, subjects were tested anyway because they were already hooked up. However, these subjects were likely to be excluded later because their performance in detecting the targets on control trials was below the preregistered 70% cut-off.

During data collection, we inspected the behavioral results of the first 10 subjects to ensure that the behavioral task worked fine. We discovered that four subjects only had unaware correct trials in one quadrant. We interpreted this as subjects using a strategy: When they saw nothing, they always guessed the same quadrant. To avoid these biases, subsequent subjects were instructed to always guess to the best of their ability about Gabor location and to avoid using any strategy when they did not see the Gabor (e.g., always responding the same quadrant). Note that these four subjects were excluded because of the preregistered criterion of at least five critical trials in each quadrant.

### EEG recording

EEG data were recorded from 64 electrodes at standard 10–20 positions, and one electrode on the tip of the nose with an Active Two BioSemi system (BioSemi, Amsterdam, Netherlands). Although 64 electrodes are sufficient to capture electrical brain activity across the head, the nose electrode was included to allow for secondary analyses with only a minimal set of electrodes and the nose as the reference (these analyses are not discussed here). An EEG cap (Electro-Cap International, Eaton, OH) was used to position the 64 electrodes together with two additional, system-specific electrodes. CMS (between PO3 and POz) served as the internal reference electrode, and DRL (between POz and PO4) as the ground electrode. These 66 positions were recorded with pin electrodes, and the tip of the nose was recorded with a flat electrode attached with an adhesive disk. Data were sampled at 512 Hz and filtered with a hardware low-pass filter at 104 Hz.

### Data analysis

The data were processed and analyzed using MATLAB (The MathWorks, Inc.), R (R Core Team, 2016), and JASP (JASP Team, 2017)*.* All physiological data were processed offline using the toolbox FieldTrip (Version 20170511) in MATLAB (Oostenveld, Fries, Maris, & Schoffelen, 2011). The behavioral analyses included all trials whereas in the EEG data analyses, some trials were excluded (see below).

In the EEG data analyses of the critical trials, Gabor onset was indexed by the photodiode triggers rather than the event marker from the presentation computer, thus eliminating any timing errors in Gabor onset. Further, the duration of the Gabor on individual trials was defined as the interval between the onset and offset triggers from the photodiodes. A critical trial was excluded if the actual duration deviated more than 2 ms from the duration mode in a condition (to account for error from a 512-Hz sampling frequency). These timing errors occurred very rarely and nonsystematically. Less than six per 1,000 trials were excluded.

Offline, continuous EEG data were high-pass filtered with a 0.1 Hz Butterworth fourth degree two-pass filter and down-sampled to 250 Hz. (We forgot to preregister these steps, but they are common steps to reduce drift and to facilitate the speed of data preprocessing.) Epochs were extracted from 100 ms before Gabor onset to 600 ms after Gabor onset. Each epoch was baseline corrected with the 100-ms interval before Gabor onset. For each subject, maximum amplitude ranges were extracted for individual epochs, and the distribution of these amplitude ranges was inspected. Individual trials were excluded if they were apparent outliers that could not have resulted from eye blinks. The exclusion thresholds were set for each individual because subjects showed substantial variability in these amplitude ranges. Critically, the inspection of trials was blind to Gabor size and trial type (critical, catch, and control) to avoid bias. If an electrode showed large amplitude ranges on many trials, it was excluded until after eye-blink detection and correction (see below). Across all subjects and all electrodes, a maximum of three electrodes had to be excluded per subject. For the relevant electrodes (the ones used for calculating VAN and LP), only one subject had one electrode that had to be excluded and interpolated. Eye blink artifacts were detected and corrected with ICA (Runica), and after this correction, the excluded electrodes were interpolated with splines. Finally, the data were baseline corrected again and rereferenced to the arithmetic mean of 64 electrodes and the nose electrode. We note that in the preregistration we stated that we would not include the nose electrode in this average reference. However, before starting the actual data preprocessing, we realized that because the nose has a frontal position, it would be valuable in detecting and correcting for eye blinks while having negligible effects on the average reference. After rereferencing, the distribution of maximum amplitude ranges was inspected again to exclude remaining outliers. As before, this inspection was adjusted individually but was blind to Gabor size and trial type to avoid bias.

### ERP analysis

Three ERPs were derived from critical trials depending on the subjective and objective rating given by each subject, as in the experiment by Koivisto and Grassini (2016). *Aware correct* were correctly localized trials rated as “I saw the stimulus clearly” or “I saw the stimulus weakly,” *unaware correct* were correctly localized trials rated as “I did not see any stimulus,” and *unaware incorrect* were incorrectly localized trials rated as “I did not see any stimulus.” In computing the ERPs, the visual quadrants were analyzed separately to avoid confounding from physical differences between the quadrants. Note that in the final sample, subjects were included only if the number of trials in each quadrant was five or more.

As defined in the preregistration, ERP amplitudes were computed from the N200 (180 to 280 ms) and P3 (350 to 550 ms) interval. For the N200 interval, contralateral electrodes were pooled (i.e., O1 for right visual field Gabors and O2 for left visual field Gabors). We considered only contralateral electrodes because cortical activity is maximal contralateral to visual stimulation (Koivisto & Grassini, 2016). For the P3 interval, P3, Pz, and P4 were pooled. For each Gabor size, difference waves were calculated by subtracting unaware correct from aware correct trials. We predicted that these difference waves would be negative over occipital electrodes in the N200 interval (VAN) and positive over parietal electrodes in the P3 interval (LP). We also extracted ERPs and mean amplitudes for the N200 from aware correct control trials. Individual ERPs to these control trials were inspected to identify subjects who did not show an apparent negativity (N200).

We conducted Bayesian hypothesis testing to determine the degree of evidence for or against the alternative hypothesis (Dienes, 2008). The Bayes factor (BF_10_) expresses the likelihood of the data given the alternative hypothesis relative to the likelihood of the data given the null hypothesis (Dienes, 2008, 2016; Wagenmakers, Marsman, et al., 2017; Wiens & Nilsson, 2017). Although the BF is a continuous measure of evidence, we adopted a common interpretation scheme (Wagenmakers, Love, et al., 2017).

The preregistered alternative hypotheses were as follows: For each Gabor size, VAN amplitudes would be negative and LP amplitudes would be positive. In addition, VAN amplitudes would be larger for large than for small Gabors. As an exploratory analysis, we also examined if LP amplitudes would differ with Gabor size. Although most of these hypotheses are directional (e.g., VAN would be negative), we conducted two-tailed Bayesian *t* tests with a Cauchy prior (0.707). This is the default prior implemented in JASP (JASP Team, 2017), as explained in a tutorial on JASP and its output (Wagenmakers, Love, et al., 2017). In within-subjects analyses, Bayesian one-sample *t* tests were used to determine if VAN and LP differed from zero for each Gabor size and if VAN and LP differed between Gabor sizes for subjects that completed both sessions (*n* = 13). In between-subjects analyses, Bayesian independent-samples *t* tests were used to determine if VAN and LP differed between Gabor sizes for subjects that completed either session (large: *n* = 24, small: *n* = 15). Because 13 of these subjects actually participated in both sessions, the observations in the two sessions are not strictly independent. Nonetheless, we conducted the independent-samples *t* tests for three reasons: First, it seems reasonable to assume that participation in one session did not affect performance in the other session. Second, the sample size was more inclusive and larger (*n* = 39) than in the within-subjects design (*n* = 13). Third, the independent-samples *t* test may be rather conservative because even within-subjects data are treated as between-subjects data—that is, individual differences are not removed from the error term.

Note that we preregistered two exploratory analyses. First, we proposed to compute the Pearson correlations between behavioral performance—as indexed by the signal-detection measures *d´* and criterion *C—*and mean VAN and LP amplitudes (Koivisto & Grassini, 2016). Second, we proposed to compute the ERPs to chance-free unaware correct trials (Koivisto & Grassini, 2016; Lamy et al., 2009). In preview, actual performance on the unaware correct trials was at chance (i.e., 25% was chance and actual performance was 26.1% for large Gabors and 28.2% for small Gabors). Therefore, the computation of ERPs for chance-free unaware correct trials was not feasible.

Raw data, scripts, and other supplementary material are available at figshare (10.17045/sthlmuni.5418967).

Below, we report the results of our analyses in which we strictly followed our preregistered exclusion criteria. Although the criteria were reasonable, the consequence was that relatively many subjects were excluded. To explore the effects of the exclusion criteria, we also analyzed the data while trying to retain as many subjects as possible (between 29 and 31 subjects). Critically, results matched those reported below (in fact, the evidential strength was stronger). These additional analyses are available via figshare.

## Results

### Behavior

Table 1 shows the descriptive statistics of the behavioral data. On average, subjects performed well (above 90% correct) on the control trials to both Gabor sizes, indicating that the subjects did the task as instructed. Critical trials had few false alarms (i.e., catch trials rated as aware) and were calibrated to approximately 50% aware. Gabor duration was about twice as long for small than for large Gabors.

**Table 1:** Descriptive statistics (mean and *SD*) of behavior data

	Large Gabors (*N* = 24)		Small Gabors (*N* = 15)		L − S	95% CI
Control: Hits (%)	97.3	(5.0)	90.8	(9.6)	6.5	[0.9, 2.1]
Catch: False alarms (%	7.7	(10.3)	7.0	(8.0)	0.7	[−5.3, 6.7]
Critical: Aware correct (%)	43.9	(11.6)	41.3	(9.2)	2.5	[−4.3, 9.3]
Critical: Unaware correct (%)	26.1	(7.5)	28.2	(9.3)	−2.1	[−8.0, 3.8]
Critical: Aware incorrect (%)	2.9	(3.8)	2.8	(4.1)	0.1	[−2.6, 2.8]
Critical: Unaware incorrect (%)	27.1	(11.6)	27.7	(10.2)	−0.5	[−7.8, 6.7]
Critical: Gabor duration (ms)	25.9	(13.5)	51.6	(15.9)	−25.7	[−35.8, −15.5]

### ERP

Figures of the grand mean ERPs for aware-correct, unaware-correct, and unaware-incorrect conditions for large and small Gabors are available via figshare. For large Gabors, the mean numbers of ERP trials were 168 (*SD* = 45), 99 (31), and 102 (45) for the aware-correct, unaware-correct, and unaware-incorrect conditions, respectively. As shown in Table 1, about 44% of the 400 critical trials were rated as aware correct and 26% as unaware correct. This corresponded to 176 aware correct trials (400 × 0.44) and 104 unaware correct trials (0.26 × 400). This means that on average, 168 / 176 = 95.5% and 99 / 104 = 95.2% were retained in the ERP analyses. For small Gabors, the mean numbers of ERP trials were 156 (*SD* = 38), 108 (37), and 104 (39) for aware-correct, unaware-correct, and unaware-incorrect conditions, respectively. Because 42% of the 400 critical trials were rated as aware correct and 28% as unaware correct (see Table 1), this corresponded to 168 and 112 trials and thus, 156 / 168 = 92.8 and 108 / 112 = 96.4% were retained in the ERP analyses, respectively. So, on average 4% to 8% of trials were excluded in the ERP analyses.

#### Visual awareness negativity

**Fig. 2:**
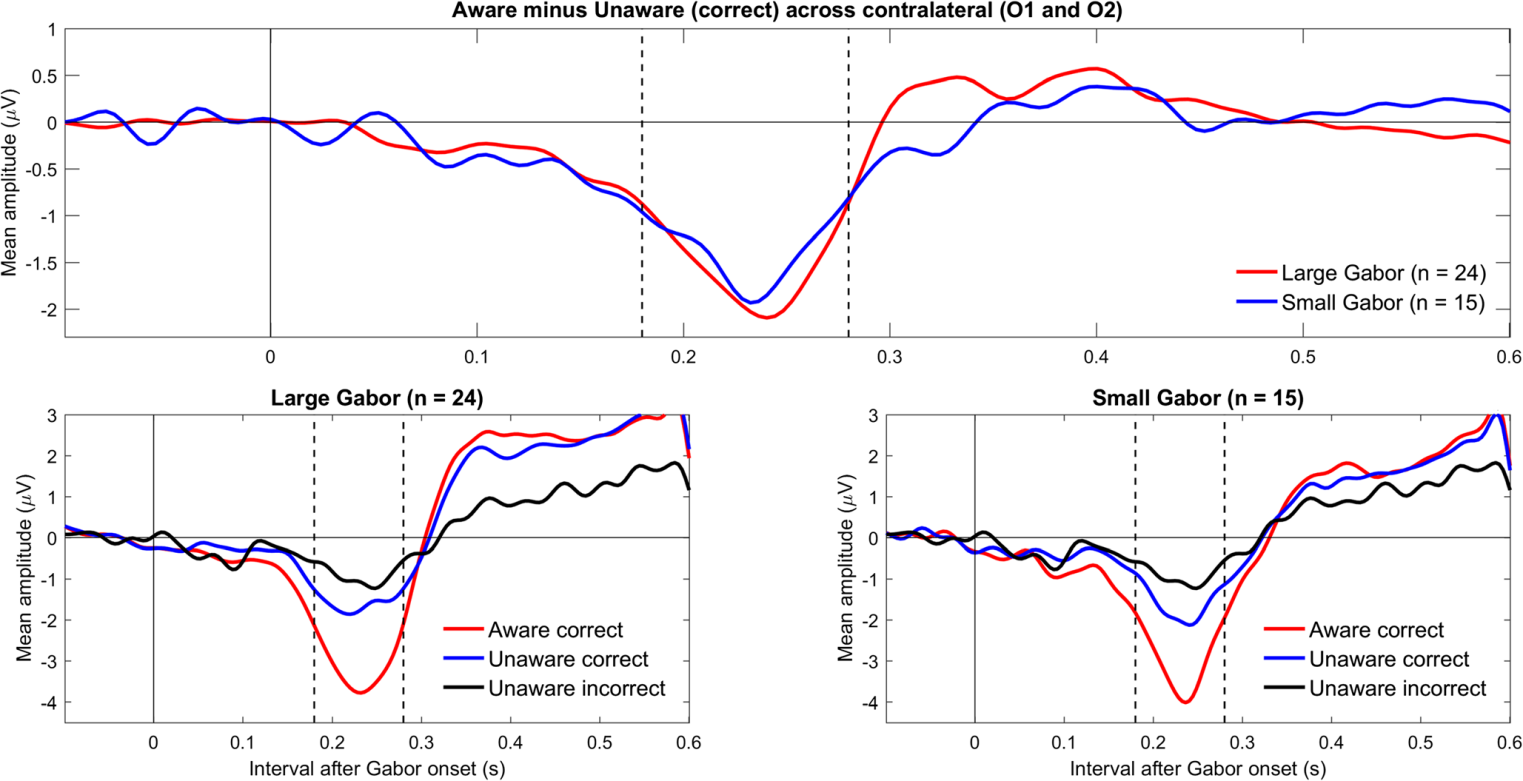
Grand mean ERPs for contralateral occipital electrodes (O1 and O2). The upper panel shows VAN for large and small Gabors. The lower panels show ERPs for aware-correct, unaware-correct, and unaware-incorrect trials for large Gabors (left) and small Gabors (right). In the plots, the data were low-pass filtered at 30 Hz. (Color figure online)

**Fig. 3:**
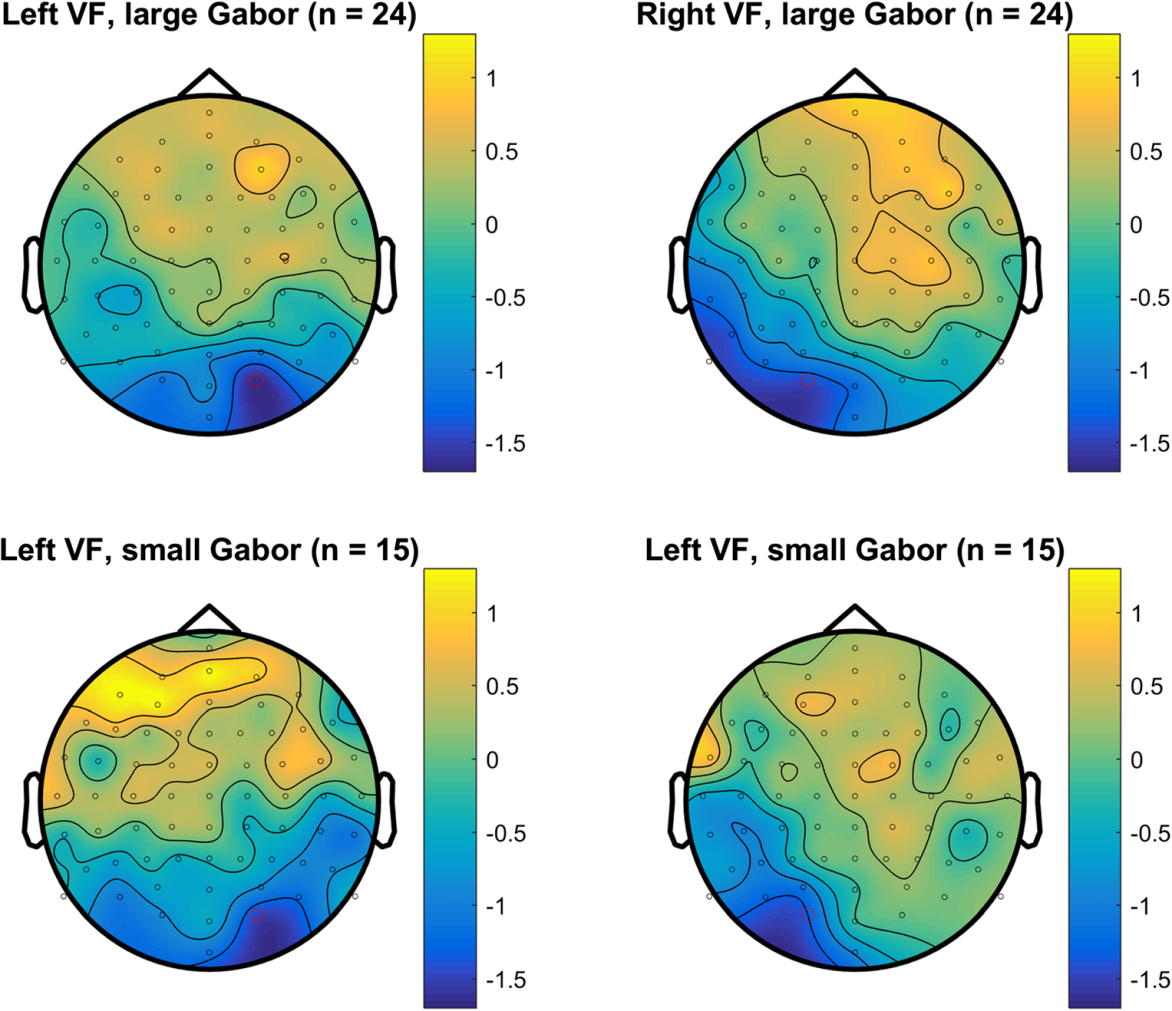
Topoplots of the mean amplitude difference for VAN between aware correct and unaware correct for left and right visual field (columns) and large and small Gabor size (rows) from 180 to 280 ms after Gabor onset. (Color figure online)

Figure **2** shows the grand mean ERPs for the different conditions for contralateral electrodes (O1 and O2) for large and small Gabors. Table **2** shows the descriptive and inferential statistics for the mean amplitudes. As shown in the top panel in Fig. **2** and supported by the Bayesian analyses, the evidence for a negativity (VAN) was very strong for large Gabors (BF_10_ = 79) and extreme for small Gabors (BF_10_ = 111) (Wagenmakers, Love, et al., 2017). By themselves, the present data suggested that the minimum plausible effect size for the VAN is −0.8 μV (with an upper limit of about −2.0 μV). Figure **3** shows the topography of the difference between aware correct and unaware correct across 180 to 280 ms after Gabor onset, separately for large and small Gabors. The negativity (VAN) can be clearly seen contralateral in relation to the stimulated visual field. As shown in Table **2**, there was no evidence for or against an effect of Gabor size on VAN (BF_10_ ≈ 0.33).

**Table 2:** Descriptive and inferential statistics for the mean amplitude differences of aware-correct trials minus unaware-correct trials

ERP condition	*N*	Mean (μV)	*SD*	95% CI	BF_10_
**VAN**					
Large Gabor	24	−1.58	1.87	[−2.37, −0.79]	79
Small Gabor	15	−1.41	1.14	[−2.04, −0.77]	111
L – S Gabor	13	−0.18		[−0.86, 0.50]	0.32
L – S Gabor	39	0.18		[−0.92, 1.27]	0.33
**LP**					
Large Gabor	24	1.35	0.92	[0.96, 1.74]	>62,000
Small Gabor	15	1.00	0.91	[0.50, 1.50]	47
L – S Gabor	13	0.18		[−0.59, 0.96]	0.31
L – S Gabor	39	−0.35		[−0.96, 0.26]	0.54

*Note.* Results of Bayesian *t* tests (two-tailed with a Cauchy prior = 0.707) of the mean amplitude difference of aware-correct trials minus unaware-correct trials. The BF_10_ expresses the likelihood of the data given the alternative hypothesis relative to the likelihood of the data given the null hypothesis. A one-sample *t* test was used for each session (*n* = 24 or *n* = 15), and a paired-samples *t* test was used for subjects who completed both sessions (*n* = 13). In an independent-samples *t* test, subjects from each session were treated as independent observations (*n =* 24 + 15 = 39). The 95% CI is the confidence interval (with a flat prior)

#### Late positivity

**Fig. 4:**
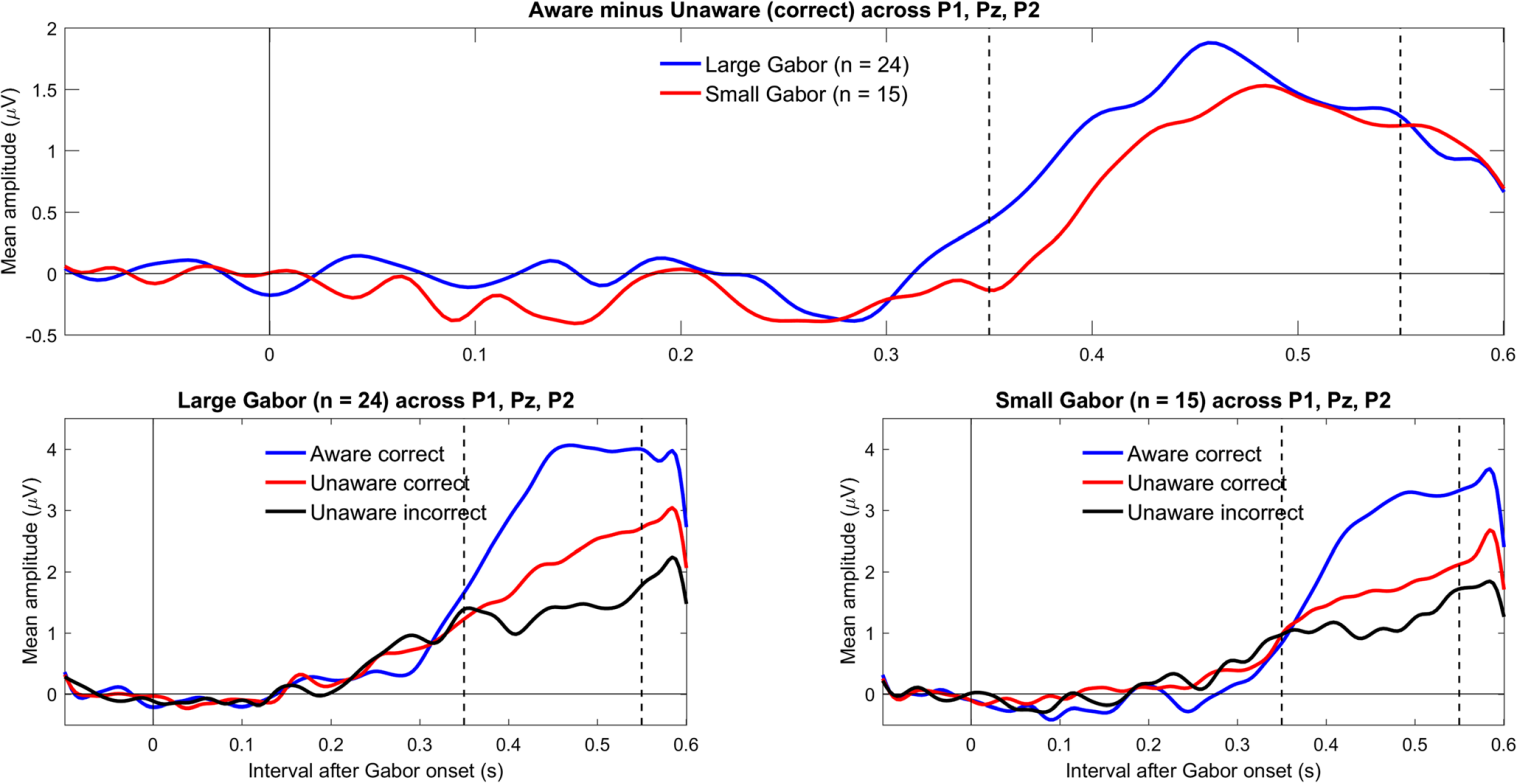
Grand mean ERPs for parietal electrodes (P1, Pz, and P2). The upper panel shows LP for large and small Gabors. The lower panels show ERPs for aware correct, unaware correct, and unaware incorrect trials for large Gabors (left) and small Gabors (right). In the plots, the data were low-pass filtered at 30 Hz. (Color figure online)

**Fig. 5:**
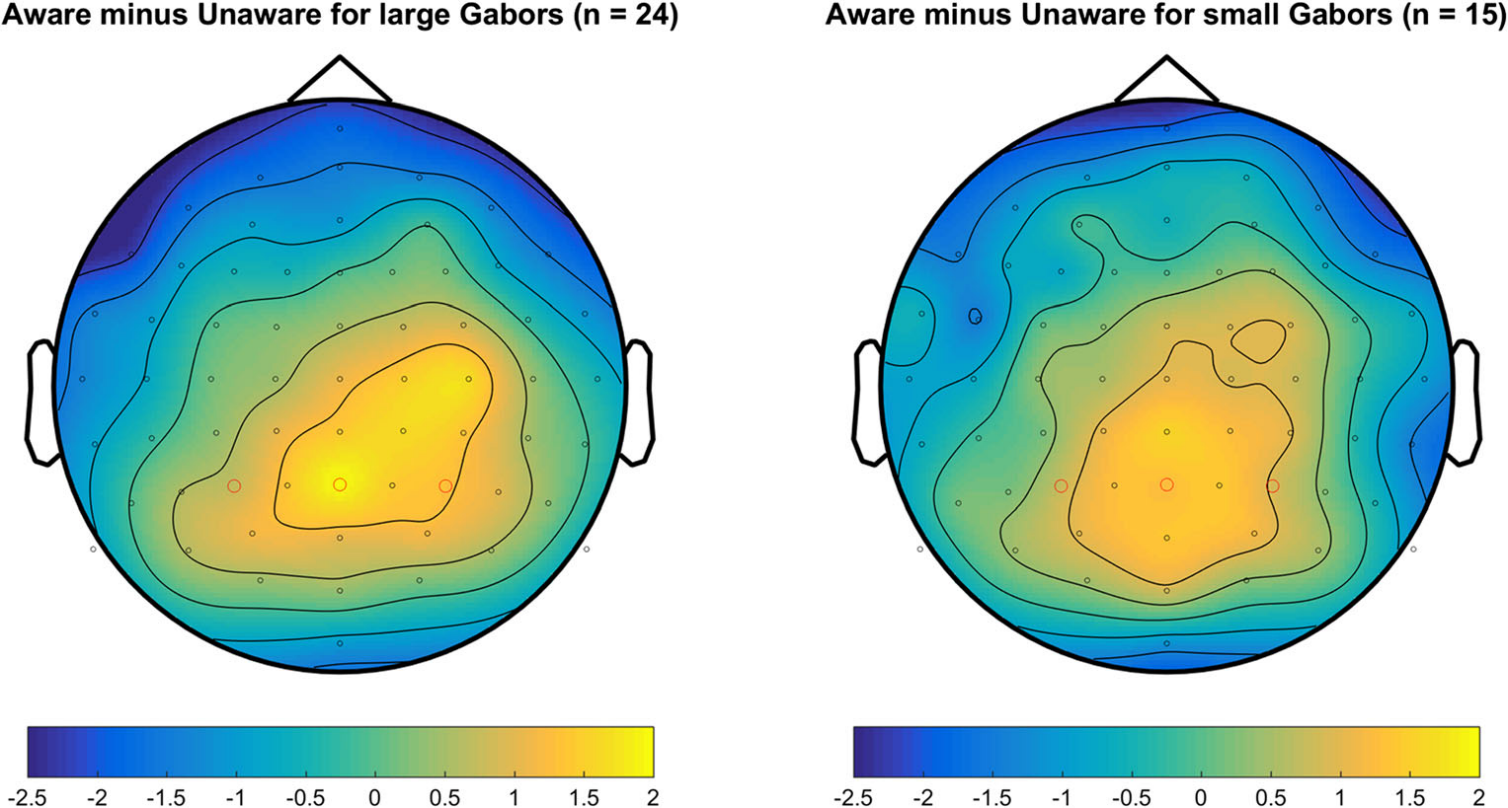
Topoplots of the mean amplitude difference for LP between aware correct and unaware correct for large and small Gabor size from 350 to 550 ms after Gabor onset. (Color figure online)

Figure **4** shows the grand mean ERPs for the different conditions across relevant electrodes (P1, Pz, and P2) for large and small Gabors. As shown in the top panel in Fig. **4** and supported by the Bayesian analyses (see Table **2**), the evidence for a positivity (LP) was extreme for large Gabors (BF_10_ > 62,000) and very strong for small Gabors (BF_10_ = 47). By themselves, the present data suggested that the minimum plausible effect size for the LP is 0.5 μV (with an upper limit of about 1.5 μV). Figure **5** shows the topography of the difference between aware correct and unaware correct across 350 to 550 ms, separately for large and small Gabors. The positivity (LP) can be clearly seen over parietal electrodes. As shown in Table **2**, there was no evidence for or against an effect of Gabor size on LP (0.31 < BF_10_ < 0.54).

### Correlations between behavior and ERP

The Pearson’s coefficients of correlation across subjects provided no evidence for a relationship between *d´* and VAN or *C* and LP for both large Gabors (*n* = 24) and small Gabors (*n* = 15). The correlation between *d´* and VAN was *r* = .00 (BF_10_ = 0.25, 95% CI [−0.38, 0.38]) for large Gabors and *r* = .10 (BF_10_ = 0.34, 95% CI [−0.39, 0.54]) for small Gabors. The correlation between *C* and LP was *r* = .10 (BF_10_ = 0.28, 95% CI [−0.30, 0.46]) for large Gabors and *r* = 0.37 (BF_10_ = 0.73, 95% CI [−0.17, 0.70]) for small Gabors.

### Differences between unaware correct and unaware incorrect

For completeness, we also explored the differences between unaware correct and unaware incorrect conditions (Koivisto & Grassini, 2016). As suggested in Fig. 2, mean amplitudes in the VAN-relevant interval were more negative for unaware correct than unaware incorrect conditions; for large Gabors (*n* = 24), mean difference = −0.99, 95% CI [−1.70, −0.29], BF_10_ = 6; and for small Gabors, mean difference = −0.70, 95% CI [−1.25, −0.16], BF_10_ = 4. Similarly, as suggested in Fig. 4, mean amplitudes in the LP-relevant interval were more positive for unaware correct than unaware incorrect conditions; for large Gabors, mean difference = 0.72, 95% CI [0.28, 1.17], BF_10_ = 14; although for small Gabors, mean difference = 0.48, 95% CI [−0.09, 1.06], BF_10_ = 1. The Pearson’s coefficients of correlation across subjects provided no evidence for a relationship between either *d´* or *C* and the mean difference (unaware correct minus unaware incorrect) in the VAN-relevant interval, or between the criterion *C* and the mean difference (unaware correct minus unaware incorrect) in the LP-relevant interval (−0.34 < *r*s < 0.37, 1.3 < BF_01_ < 4.0).

## Discussion

The main findings were that for two different Gabor sizes, results showed very strong to extreme evidence for both VAN and LP. Because VAN was observed for both Gabor sizes, these results provide strong support for the claim that VAN is an early neural correlate of awareness. However, there was only inconclusive evidence for an effect of Gabor size on VAN and LP, indicating that the present findings do not allow any conclusions about an effect of Gabor size.

We conducted a preregistered study that improved on the design of previous studies (Koivisto & Grassini, 2016; Lamy et al., 2009; Salti et al., 2012). First, previous studies pooled all trials irrespective of the quadrants (i.e., weighted), whereas we averaged the quadrants separately before computing the ERPs (i.e., unweighted). This procedure avoided confounds from differences in visual field stimulation. For example, the N1 (between about 120 and 220 ms) is smaller to upper than lower visual field stimulation (Capilla et al., 2016). Second, we preregistered the study in as much detail as possible (Eklund & Wiens, 2017, osf.io/4a4ur). This increases the evidential strength of the present results because without preregistration, ERP studies in general may be criticized for allowing substantial flexibility in how the data can be analyzed (John, Loewenstein, & Prelec, 2012; Luck & Gaspelin, 2017; Simmons, Nelson, & Simonsohn, 2011). Third, because we validated Gabor onset and duration with photodiode measurements, timing errors in the results were avoided.

The present findings challenge the previous null results with respect to VAN (Lamy et al., 2009; Salti et al., 2012). Koivisto and Grassini (2016) proposed several potential explanations for these null findings: small stimuli, backward masking, and pooling of ipsilateral and contralateral electrodes. Another possible explanation is that stimulus eccentricity was much larger in the previous studies (4°) than in the Koivisto and Grassini study (0.3°). Stimulus eccentricity affects the amplitude of the early visual evoked potentials even if cortical magnification is taken into account. For example, N1 amplitudes decrease with eccentricity (Capilla et al., 2016). Another explanation is that spatial frequency was lower in previous studies (three lines spanning a square of 1.5°) than in the Koivisto and Grassini study (10 c/deg), and N1 amplitudes increase with spatial frequency of high-contrast Gabors (Mihaylova, Hristov, Racheva, Totev, & Mitov, 2015). Notably, a recent study showed that VAN varies with the calibration threshold such that VAN was observed at the detection threshold but not the identification threshold (Koivisto, Grassini, Salminen-Vaparanta, & Revonsuo, 2017). In the study, awareness was calibrated separately to the detection and identification thresholds with the same subjective rating scale: “I did not see any stimulus,” “I saw something (but could not identify the stimulus),” “I saw the stimulus almost clearly (and could identify it),” and “I saw the stimulus clearly (and could identify it).” In a detection task, stimuli were calibrated to be rated as “I did not see any stimulus” in about 50% of the trials (not aware of the stimulus). In a separate identification task, stimuli were calibrated to be rated as “I did not see any stimulus” or “I saw something (but could not identify the stimulus)” in about 50% of the trials (not aware of the identity of the stimulus). Results showed that VAN was present at the detection threshold (i.e., for detected minus undetected trials) but not at the identification threshold (i.e., for identified minus unidentified trials). These results imply that whether or not VAN is observed depends on the calibration threshold. In previous studies (Lamy et al., 2009; Salti et al., 2012), subjects had to detect the stimulus in any of four locations without having to identify the stimulus. Arguably, this task captures the detection threshold and VAN would have been expected. Last, whereas Salti et al. (2012) excluded unsure trials, both Koivisto and Grassini (2016) and the present study combined unsure trials with clearly aware trials, mainly because there were only few clearly aware trials. Because combining unsure trials with clearly aware trials weakens the difference in awareness between aware and unaware trials, any findings would represent a conservative estimate of VAN. Accordingly, a stronger VAN would have been expected in Salti et al. (2012) because unsure trials were excluded. However, because Lamy et al. (2009) compared trials in which subjects reported that they saw the target with those in which they guessed its location, subjects may have been inclined to report awareness only if they clearly saw it. Consistent with this interpretation, subjects performed much better than chance on unaware trials (about 50% to 60%, whereas chance level was 25%). If the unaware trials contained many trials in which subjects were actually aware, the difference in awareness between aware and unaware trials might be too small to produce VAN. Taken together, any of the abovementioned explanations or a combination thereof may account for the previous null findings.

In line with predictions by Koivisto and Grassini (2016), we hypothesized that VAN would be stronger for large than small Gabors. Results showed only inconclusive evidence for or against an effect of Gabor size. Therefore, the present results do not permit any conclusions about an effect of Gabor size. However, the potential lack of an effect may be expected given the experimental design: Because larger Gabors are more easily detected, other stimulus parameters such as duration or contrast need to be adjusted to reach awareness thresholds. In the present study, duration was adjusted to each subject’s awareness threshold, separately for large and small Gabors. Small Gabors were shown for a mean duration of 52 ms, whereas the large Gabors were shown for a mean duration of 26 ms (see Table 1). Because small Gabors were shown about twice as long as were large Gabors, the summation of size and duration may have led to comparable effects on the ERPs. Indeed, the ERPs to the aware-correct and unaware-correct conditions were comparable for large and small Gabors (see Figs. 2 and 4). Notably, confounding of various parameters occurs by necessity in this typical experimental design. Because size (Mihaylova et al., 2015; Pfabigan, Sailer, & Lamm, 2015), contrast (Souza et al., 2013), as well as duration affect the amplitude and latency of visual evoked potentials, there is a built-in confound when using this design.

To investigate the effect of size without these confounding effects, a design should be used that does not rely on low contrast. For example, masking, attentional blink, change blindness, or bistable perception could be used to measure VAN (Koivisto & Revonsuo, 2010). In these tasks, the Gabor size can be varied while controlling for contrast and duration.

Importantly, previous studies showed that ERPs over occipital electrodes are sensitive to effects of stimulus size. When circles and squares of different sizes (1.5°, 5°, and 8°) were shown at fixation, P1 and N1 amplitude increased slightly with stimulus size (Busch, Debener, Kranczioch, Engel, & Herrmann, 2004). When plus and minus signs of different sizes (0.5°, 1.8°, and 4.5°) were shown at fixation, P1 and N1 increased with stimulus size (Pfabigan et al., 2015). In one study, Gabors were shown at fixation at a contrast three times above detection threshold, and size (height and width) and spatial frequency were manipulated (Mihaylova et al., 2015). Height and width were shown at 0.15°, 0.29°, 0.58°, 1.17°, and 2.33°, and spatial frequency was shown at 1.45 c/deg, 2.9 c/deg, and 5.8 c/deg. Results suggested that size (height and width) increased P1 amplitudes, particularly so at high spatial frequency. In contrast, height (but not width) increased N1 amplitudes, but only at high spatial frequency. Because of these findings, size might have an effect on the early N1 and P1. However, a potential concern is that ERPs might not be sensitive enough to detect a size effect due to the relatively small difference in cortical area between a large and a small stimulus. If so, a more sensitive measure might be needed such as local field potentials from multi-unit recordings in an animal model.

If the amplitude of feedback activation from higher visual areas to the corresponding receptive fields in V1 and V2 increases with Gabor size, VAN amplitude, as an index of recurrent processing, should increase because a larger area of cortex is activated. If so, this finding would support the view that higher visual areas feed back into lower visual areas that act as “active blackboards” (Bullier, 2001). Such a finding would also be consistent with other research that suggests that visual awareness is gradual in its nature (Andersen et al., 2016; Koivisto et al., 2016; Sandberg, Timmermans, Overgaard, & Cleeremans, 2010).

As in many other studies, subjects in the present study had to report their awareness on each trial. Because this design confounds visual awareness and the subjective report of this awareness, phenomenal consciousness is confounded with access consciousness (Block, 2007). Therefore, the present findings do not disentangle if VAN is the correlate of phenomenal consciousness or just a prerequisite to access consciousness (Dehaene et al., 2006). Importantly, new tasks avoid this problem by dissociating the neural activity enabling phenomenal consciousness from activity enabling report (Tsuchiya, Wilke, Frässle, & Lamme, 2015). These studies contrast conditions in which subjects are aware and unaware of a stimulus without subjects making a planned motor response. For example, Koivisto et al. (2016) used a go/no-go task in which subjects responded with a keypress when being aware of the stimulus (aware go) and withheld their response when not being aware of the stimulus. Because subjects also performed the task with reverse response conditions (unaware go), this design disentangles effects of response requirements from awareness. Results by Koivisto et al. suggest VAN rather than LP as the neural correlate of visual awareness. Frässle, Sommer, Jansen, Naber, & Einhäuser (2014) used a binocular rivalry task in which stimuli were either dynamic (inducing optokinetic nystagmus) or static with different luminance (inducing change in pupil size). While the dominant percept naturally switched between left and right visual input, subjects either actively indicated their dominant percept with a button press or just passively watched. The objective measures were used to track the dominant percept, and the active task was used to verify and validate the robustness of the objective measure. Their finding suggest occipital and parietal activation related to subjects’ visual experience in the passive viewing task and additional frontal activation in the active response task (Frässle et al., 2014). Indeed, several studies with nonresponse paradigms suggest that activity in the posterior cortical areas rather than the prefrontal cortex correlates with visual awareness (for a review, see Koch et al., 2016).

To conclude, we successfully replicated and extended previous studies. The present results with two different Gabor sizes provide very strong evidence for VAN as an early neural correlate of awareness. However, the results provide no evidence for or against an effect of Gabor size on VAN or LP. Because by design size is confounded with changes in other parameters (e.g., duration) in the present paradigm, the summation of these effects may be expected to produce similar effects on VAN and LP.
